# Compatibility of Commonly Used Active Pharmaceutical Ingredients in a Ready-to-Use Oral Suspending Vehicle

**DOI:** 10.3390/pharmaceutics15102388

**Published:** 2023-09-26

**Authors:** Mercedeh Mansourian, Eli Dijkers, Carolina C. V. Silva, Hudson C. Polonini

**Affiliations:** Fagron BV, 3065 WB Rotterdam, The Netherlands; mersedeh.mansourian@fagron.com (M.M.); eli.dijkers@fagron.com (E.D.); carolina.costa.vicente.silva@fagron.com (C.C.V.S.)

**Keywords:** SyrSpend^®^ SF PH4, oral suspensions, compounding

## Abstract

The present study aimed to evaluate the stability of active pharmaceutical ingredients (APIs) from different pharmacological classes in a compounded oral suspending vehicle. Oral suspensions of amoxicillin trihydrate (50 mg/mL), clozapine (25 mg/mL), indomethacin (5.0 mg/mL), levodopa/carbidopa (10.0/2.5 mg/mL), levothyroxine sodium (T4, 25 µg/mL), lomustine (4.0 and 10.0 mg/mL), methyldopa (25 mg/mL) and procarbazine (10.0 mg/mL) were formulated in SyrSpend^®^ SF PH4 and the stability was monitored for up to 90 days, except for amoxicillin trihydrate, which was evaluated for 30 days only. The APIs’ stability was determined by measuring percent recovery using stability-indicating high-performance liquid chromatography (HPLC or UHPLC) or titration (amoxicillin trihydrate only). The stability of amoxicillin trihydrate, clozapine, indomethacin and levodopa/carbidopa were studied at both refrigerated (2–8 °C) and room temperature (20–25 °C). Lomustine, procarbazine, and methyldopa were studied at refrigerated temperature only. Our data demonstrated promising stability for the compounded suspensions containing various APIs, investigated in SyrSpend^®^ SF PH4, as all APIs exhibited stability throughout the study duration and met content uniformity criteria. These findings lead to the conclusion that the tested compounded oral suspensions present a viable approach for creating personalized, age-appropriate formulations. The capacity to ensure dose consistency and stability using APIs from diverse pharmacological classes renders them suitable choices for both pediatric and geriatric patients.

## 1. Introduction

Oral administration is the main route of drug delivery. Advantages include versatility, ease of swallowing, avoidance of administration discomfort, and increased patient compliance [1]. However, children and older adults differ in many aspects from the other age groups and require particular considerations regarding pharmacokinetics, formulation composition, and dosage forms [2]. Across the pediatric groups, organ size and function rapidly change, as do body composition, cellular function, and metabolic activity, potentially leading to pharmacokinetics differences [3]. In geriatric patients, pharmacokinetics is strongly influenced by comorbidity, reduced organ function or polypharmacy [4].

In addition, the final medicinal product includes active pharmaceutical ingredients (APIs) and excipients [5]. However, excipients accepted in adult formulations may not be suitable for specific groups such as pediatrics and geriatrics [2,6]. Neonates may not be able to clear an excipient in the same manner as adults due to their physiological and developmental immaturity [7]. Similarly, high sodium intake may disturb the electrolyte balance, leading to water retention and increasing the risk of cardiovascular diseases in older patients [8].

Furthermore, although most oral processes are present from birth (rooting, lip, lateral tongue, mouth opening, biting, and emerging chewing behaviors), the main issue of oral administration is related to the ability to swallow the medication effectively [9]. Many patients find it difficult to swallow tablets and hard capsules, and this difficulty is especially prevalent in pediatric and geriatric patients [10,11]. Medicines commonly need to be split, crushed, or processed otherwise, and all these can lead to inaccurate dosing and potentially threaten patient safety [12]. Thus, age-appropriate liquid formulations are desirable to accurately deliver the right dose and match the specific physiological characteristics of both young and older patients [13,14,15]. However, formulating oral liquid dosage forms can be challenging due to the lack of data on the physicochemical stability of such preparations in the context of compounding pharmacies.

In this context, SyrSpend^®^ SF PH4 is a ready-to-use suspending vehicle based on food starch. It allows quick and easy compounding by pharmacists. SyrSpend^®^ SF is free of sucrose, alcohol, sorbitol, or any other hazardous excipients [16]. SyrSpend^®^ SF PH4 NEO is a dry version of SyrSpend^®^ SF PH4 liquid and preserved with 0.2% potassium sorbate, making it more suitable for younger children, including neonates.

Moreover, drug shortages have become a global health concern affecting patients’ treatment options in low, middle, and high-income countries. All commonly used drugs, such as antimicrobials, analgesics, and cardiovascular drugs, are liable to shortage [17]. As most of these medicines can be compounded into personalized oral suspensions by (hospital) pharmacists, it is crucial to assess the physicochemical stability of those formulations.

Therefore, this study was designed to evaluate the stability of commonly used APIs (amoxicillin trihydrate, clozapine, indomethacin, levodopa/carbidopa, levothyroxine sodium, lomustine, methyldopa, and procarbazine) compounded with SyrSpend^®^ SF PH4 to determine their beyond-use dates. 

## 2. Materials and Methods

### 2.1. Reagents, Reference Standards, and Equipment

All APIs (Table 1), SyrSpend^®^ SF NEO and SyrSpend^®^ SF PH4 were supplied by Fagron (São Paulo, Brazil). HPLC-grade reagents were procured from Vetec (Rio de Janeiro, Brazil). Ultrapure water, with an 18.2 MΩ cm resistivity at 25 °C and less than 10 ppb total organic carbon, was used throughout the experiments and produced using an AquaMax-Ultra 370 Series system (Young Lin, Anyang, Republic of Korea). The reference standards were acquired directly from the United States Pharmacopeia (USP, Rockville, MD, USA). Immediately before use, all mobile phases were filtered through a 0.45 mm filter membrane (RC-45/15 MS; Chromafil, Düren, Germany) and degassed for 30 min in an ultrasonic water bath (model 1600A; Unique, Indaiatuba, Brazil). All analytical balances and volumetric glassware were calibrated. For indomethacin, UHPLC analyses were performed in a qualified and calibrated Thermo Scientific (Waltham, MA, USA) equipment model Vanquis with software controller Chromeleon 7, version 7.3. HPLC analyses were performed in qualified and calibrated Young Lin equipment with a software controller Clarity version 8.1 (for levodopa/carbidopa, levothyroxine sodium and lomustine) or an Agilent (Santa Clara, CA, USA) equipment model 1260 Infinity with software controller OpenLab CDS version 2.7 (for clozapine, methyldopa and procarbazine).

### 2.2. Titration

The amoxicillin trihydrate stability was assessed using the official USP-National Formulary (USP-NF) method <425> Iodometric assay—antibiotics. In short, the amoxicillin in SyrSpend^®^ SF PH4 was diluted to a 1 mg/mL preparation in water. To 2.0 mL of the Standard Preparation and of the Assay Preparation, in respective flasks, 2.0 mL of 1.0 N sodium hydroxide was added, mixed by swirling and allowed to stand for 15 min. To each flask 2.0 mL of 1.2 N hydrochloric acid and 10.0 mL of 0.01 N iodine volumetric solution (VS) were added. Immediately the stopper was added and allowed to stand for 15 min. The mixture was titrated with 0.01 N sodium thiosulfate until the endpoint was approached; then, one drop of starch iodide paste was added, and the titration was continued until the blue color disappeared.

### 2.3. Chromatographic Conditions

The official USP method for each API was followed during the chromatographic assessments with minor adjustments if needed. Table 2 details the mobile phase, employed standard diluents, injection volumes for each API, and the used columns.

### 2.4. Validation of Chromatographic Methods 

The acceptance criteria and methods were established following the guidelines provided by the USP protocols [18] and the International Conference on Harmonization (ICH) [19].

The specificity of the methodology was determined by running chromatographic analyses on a standard solution, a blank solution of SyrSpend^®^ SF PH4 (liquid or NEO versions), and a blank solution of the mobile phase/diluent. The acceptance criterion was defined as a percentage of a discrepancy between the peak areas lower than 2%. Moreover, the specificity of the methodology was confirmed by comparing standard chromatograms with and without the matrix. All analyses were conducted in triplicate.

To assess precision, the test was designed to evaluate the degree of variation among a series of measurements obtained by the same analyst (repeatability) and between two analysts and over 2 days (within-laboratory variations, intermediate precision) for solutions of the API at working concentrations. Repeatability was determined by consecutively analyzing six replicates by a single analyst in a single day. Intermediate precision was also performed on six replicates, but over 2 days, by different analysts. An injection precision of more than 95% (coefficient of variation, CV) was considered acceptable.

The accuracy of the methodology was determined through spike-recovery of the SyrSpend^®^ SF PH4 matrix, diluted within the range used for final sample measurements, and within the range of the corresponding calibration curves. The recovery percentage was calculated from the concentration measured relative to the theoretical concentration spiked.

For linearity, the test was conducted by constructing three genuine replicates of standard curves from three separate samplings, each consisting of the API concentrations of 70–130% of work concentrations. This was conducted to evaluate the linear relationship between the analyte’s concentration and the obtained areas, in the presence of the SyrSpend^®^ SF PH4 matrix. To accomplish this, the data for each concentration range of the curve were evaluated using analysis of variance (ANOVA) and subjected to the least squares method to determine the correlation coefficient of the calibration curve.

The limit of detection (LOD) and limit of quantification (LOQ) were determined from three standard calibration curves of the API in the presence of the SyrSpend^®^ SF PH4 matrix and were calculated as indicated in Equations (1) and (2), respectively:

(1) $$LOD=S\frac{3}{a}$$

(2) $$LOD=S\frac{10}{a}$$

The slope of the calibration curve, represented by ‘a’, and the standard deviation of the y-intercept, represented by ‘S’. The accuracy of the LOD and LOQ values was confirmed by analyzing chromatograms generated from solutions with concentrations at or below their respective limits.

### 2.5. Formulating the Suspensions

The suspensions containing raw powders were prepared in accordance with the following standardized procedures, depending on the vehicle.

In the case of clozapine, no raw pharmaceutical material could be obtained, and a licensed medication (Leponex^®^ Mylan) was used instead. The required quantity of tablets was calculated, and the tablets were ground into a fine powder using a mortar and pestle before they were used in an identical way as the raw pharmaceutical material to compound the suspensions.

#### 2.5.1. SyrSpend^®^ SF PH4 NEO

The necessary quantity of the API was calculated to achieve the desired total amount. Accurate weighing and/or measuring of API and SyrSpend^®^ SF PH4 NEO were carried out in the second step. Next, all ingredients were triturated and mixed using geometric techniques. Subsequently, purified water was added geometrically until the required amount was reached. To ensure a homogeneous mixture, the formulation was passed through a sifter. Finally, the prepared product was packaged in an amber bottle and appropriately labeled. 

#### 2.5.2. SyrSpend^®^ SF PH4 Liquid

Firstly, the total amount of each ingredient required was calculated and then weighed with precision. The API was then triturated until it formed a fine powder and a small amount of SyrSpend^®^ SF PH4 (liquid) was added to create a uniform paste. Gradual additions of SyrSpend^®^ SF PH4 (liquid) were made until the desired volume was nearly reached, with thorough mixing after each addition. Finally, the defined volume was reached by adding the remaining SyrSpend^®^ SF PH4 (liquid) and mixing thoroughly. The T = 0 concentration was determined, and the suspension was divided into two halves. One part was stored at the USP-recommended refrigerated temperature (2–8 °C), the other at room temperature (20–25 °C) throughout the study, except in the case of levothyroxine and lomustine, which were conducted at refrigerated temperature only. Temperature and humidity were monitored using a calibrated, digital thermohygrometer (Incoterm, Porto Alegre, Brazil), and both bottles were stored and protected from light. The bottles were shaken thoroughly before sampling.

### 2.6. Forced-Degradation Studies: Stability-Indicating Characteristics

The ability of the chromatographic methods to detect any potential degradation products that may arise during storage of the oral suspension was evaluated by subjecting the samples to various stress conditions including dilution in 0.1 M HCl, dilution in 0.1 M NaOH, exposure to UV light at 365 nm for 24 h, dilution in peroxide (H_2_O_2_) 35% (*v*/*v*) at 25 °C and heating at 70 °C for 24 h. Prior to injection into the chromatographic system, the stock solutions underwent sonication for a duration of 10 min and were subsequently filtered through regenerated cellulose syringe filters with a 0.45 µm pore size. Any additional peaks identified in the resulting chromatograms were appropriately labeled. The degree of separation between the degradation products and API peaks was determined and a resolution of at least 1.5 was required for complete separation.

### 2.7. Stability Study

The stability of API in SyrSpend^®^ SF PH4 was determined by assaying API samples at predetermined time points using HPLC/UHPLC or titration (amoxicillin trihydrate only). The samples were manually shaken for 1 min to simulate patient dosing. Volumetric aliquots were withdrawn from the middle of the bottles without contacting the inner surface of the bottle and diluted to obtain working solutions (Table 2). The samples were taken at several time points, including baseline: T = 0, 7, 14, 30, 60, and 90 days. The samples were diluted, sonicated for 10 min, passed through regenerated cellulose syringe filters with 0.45 μm pore size, and then injected into the HPLC/UHPLC system. All samples were immediately assayed six times at each time point. In the case of levodopa/carbidopa, both APIs were analyzed separately. The results were expressed as the percent recovery at T = 0 ± standard deviation (SD). As a critical aspect of our methodology, we conducted visual inspections before each sampling event to assess the physical stability and homogeneity of the oral suspensions. These inspections involved a thorough examination of the suspensions for any signs of caking, flocculation, macroscopically visible crystal growth, odor generation, phase separation, precipitation, or turbidity.

## 3. Results

The results of the method validation are presented in Table 3. As can be seen in Table 3, specificity, precision, accuracy and linearity met the acceptance criteria. 

The stability-indicating studies were used to confirm the full validation and adequacy of the methods. API decomposition was identified through chromatographic analysis after forced degradation of the APIs. The results of the stability-indicating study are presented in Table 4. The analysis revealed that all APIs, except for levodopa, showed various decomposition patterns under different stress conditions. 

The chromatograms of Methyldopa displayed no significant differences when subjected to acid treatment (0.1 M HCl) compared to the non-treated controls. The chromatograms of all other APIs exhibited changes when exposed to either base or acid conditions. UV exposure significantly influenced the chromatographic response of clozapine, indomethacin, lomustine, and procarbazine. When exposed to heat (70 °C), only levodopa exhibited minimal non-significant changes. H_2_O_2_ exposure impacted all APIs except for levodopa, carbidopa, and methyldopa.

It is worth mentioning levodopa stood out as the only API that remained (almost) unaffected by all applied various stress conditions. This is consistent with what we had seen in our previous stability study with levodopa 5.0 mg/mL + carbidopa 1.25 mg/mL in SyrSpend^®^ SF PH4 [20]. As amoxicillin trihydrate’s experimental procedure strictly adhered to the USP-NF method <425>, a stability-indicating study was, therefore, not needed [18].

**Figure 1:**
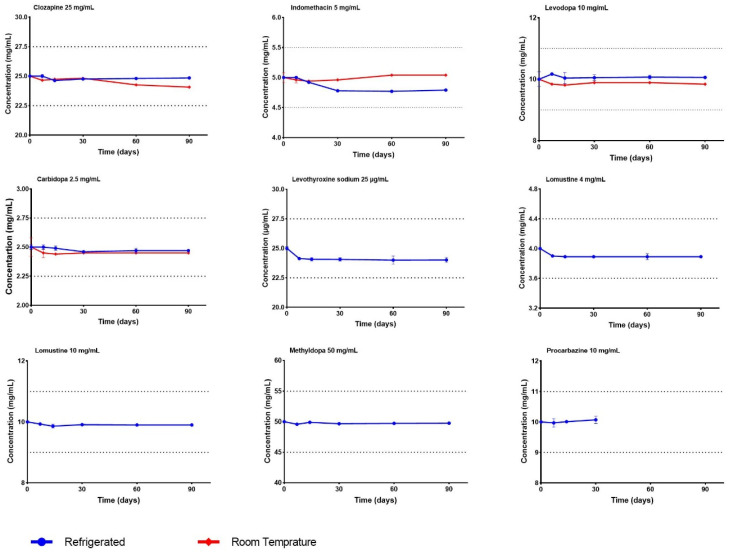
Stability of the selected APIs in SyrSpend^®^ SF PH4 throughout the study.

Table 5 presents the stability results, represented as the percentage of recovery relative to the initial sampling time. To meet the stability criteria established by international pharmacopeias1, the relative percentage of recovery should fall between 90 and 110%. Figure 1 visually illustrates the compatibility of the APIs studied in SyrSpend^®^ SF PH4. 

Right before sampling, the suspensions underwent visual inspection at each sampling time to confirm their homogeneity and physical stability. No caking, flocculation, macroscopically visible crystal growth, odor generation, phase separation, precipitation, or turbidity were seen during the entire study for any of the suspensions.

## 4. Discussion

### 4.1. Amoxicillin

The stability of amoxicillin trihydrate oral suspension in SyrSpend^®^ SF PH4 was investigated under storage conditions of 2–8 °C and 25 °C for 30 days. The results demonstrated that the formulation remained stable throughout the study duration in both temperature conditions. At 25 °C, a decline of 5.62% was observed after 30 days, although no accompanying physical changes were noted.

The study ran for 30 days, as the typical amoxicillin treatment duration is generally limited to 2 weeks only. The stability found in our study is in line with that reported by Allen and Lo et al. In their study, a 7.5% decrease in stability was observed over 30 days, where most of the loss in content occurred within the first 10 days at room temperature for amoxicillin, repackaged in unit dose containers [21].

These results confirm that an amoxicillin oral suspension in SyrSpend^®^ SF PH4 NEO can be assigned a beyond-use date of 30 days when stored at refrigerated or room temperature.

### 4.2. Clozapine

Clozapine in SyrSpend^®^ SF PH4 was stable for at least 90 days, regardless of the storage temperature. Walker et al. also showed prolonged stability of clozapine in an oral suspension prepared in a 1:1 mixture of methylcellulose gel 1% and syrup, which was stored in amber glycol-modified polyethylene terephthalate (PET-G) bottles [22]. Their study demonstrated that the suspension had a drug content above 95% for over 120 days at both 4 °C and 25 °C. Minimal loss of concentration, less than 2%, of clozapine on day 90 supports the compatibility of clozapine with SyrSpend^®^ SF PH4 (liquid) at refrigerated and room temperature over 90 days.

### 4.3. Indomethacin

Our current results demonstrated that indomethacin suspension in SyrSpend^®^ SF PH4 remained stable for at least 90 days. This demonstrated an improved beyond-use date compared to earlier performed studies. Yanina de Lafuente examined an indomethacin 0.2% oral suspension prepared from a licensed injectable and found stability of only 17 days at room temperature [23]. Stewart et al. evaluated six extemporaneous formulations, ranging from 0.25 to 5 mg/mL, and found relatively rapid degradation of indomethacin in four formulations, while all formulations exhibited issues such as caking or color changes [24].

### 4.4. Levodopa/Carbidopa

Our group previously evaluated the compatibility of levodopa 5.0 mg/mL + carbidopa 1.25 mg/mL in SyrSpend^®^ SF PH4 under two different storage conditions (2–8 °C and 20–25 °C). In the current study, a longer BUD was found for carbidopa (2.5 mg/mL) in SyrSpend^®^ SF PH4 (liquid), compared to our previous study. The results showed that the stability of the suspension was maintained for at least 90 days under storage conditions for both levodopa and carbidopa in this suspension. Pappert et al. previously reported instability for a lower concentration of levodopa/carbidopa (1.0/0.25 mg/mL) in an unbuffered aqueous system, resulting in concentration losses of up to 60%, when stored at room temperature. For the duration of the study (7 days), levodopa/carbidopa remained stable in refrigerated and frozen conditions [25]. More recently, Nahata et al. also investigated a levodopa/carbidopa oral suspension using an equal mixture of Ora-Sweet^®^ and Ora-Plus^®^ at a lower concentration than the current study. Their suspension was stable for 42 days when refrigerated [26]. The added value of adding ascorbic acid to increase the stability remains unclear. Nahata et al. found that ascorbic acid increased the decomposition rate and caused a darker yellow coloration during prolonged storage most likely due to the decomposition of ascorbic acid, whereas Pappert et al. found the ascorbic acid to be beneficial [26]. 

SyrSpend^®^ SF PH4 demonstrated the ability to preserve both the potency and physical characteristics of the levodopa/carbidopa suspension for an extended period, especially with higher concentrations of both APIs. During the 90-day storage period, the maximum loss of carbidopa was only 1%, and there was no significant loss of levodopa in either formulation.

### 4.5. Levothyroxine Sodium (T4)

Levothyroxine sodium 25 µg/mL suspensions, formulated with SyrSpend^®^ SF PH4 as the vehicle, demonstrated to be stable for 90 days when stored under refrigerated conditions. In an earlier study, using tablets and without any preservative, the suspension showed at best about 6% loss over an eight-day period.10 Oral suspensions of levothyroxine sodium formulated in a 1:1 mixture of Ora-Sweet^®^ and Ora-Plus^®^ or simple syrup NF and 1% methylcellulose (1:10) conducted by Nahata et al. exhibited only 14 days stability at 4 °C and only 7 days stability at 25 °C [27].

### 4.6. Lomustine

SyrSpend^®^ SF PH4 was used to formulate lomustine at two concentrations (4 and 10 mg/mL). The results showed that at both concentrations over 97% of the initial concentration was maintained for a minimum of 90 days when stored at a refrigerated temperature. No suitable comparative studies for lomustine could be identified in the literature.

### 4.7. Methyldopa

Our study demonstrated that methyldopa, when formulated in SyrSpend^®^ SF PH4, exhibited stability for at least 90 days at refrigerated temperature. This is much in line with the dosage forms created from commercial injections containing 50 mg/mL of methyldopa hydrochloride in simple syrup that proved to be stable for 99 days when stored at 24 °C [28].

### 4.8. Procarbazine

Procarbazine 10 mg/mL in SyrSpend^®^ SF PH4 showed no loss in content in the first 30 days when stored at 2–8 °C. There was a slight increase in procarbazine degradation, with about 5% loss on day 60. Bravo et al. previously also evaluated the stability of 10 mg/mL procarbazine in SyrSpend^®^ SF PH4 in oral suspension prepared from licensed capsules [29]. They observed similar stability of procarbazine in SyrSpend^®^ SF PH4 for the duration of their study (50 days). The study protocols are very similar, except for the fact that Bravo et al. added more excipients such as citric acid as a buffering agent, povidone K30 as a suspending agent, and taste masking agents in their formulation [29].

## 5. Conclusions

The obtained results indicate that SyrSpend^®^ SF PH4 is compatible with all eight APIs tested and displayed a beyond-use date similar to what has been studied in other vehicles or better. These findings, therefore, support the suitability of SyrSpend^®^ SF PH4 as a vehicle for compounding a broad range of APIs for compounded oral liquid medication tailored to the needs of various patient groups, such as pediatric or elderly patients.

## Figures and Tables

**Table 1:** List of active pharmaceutical ingredients (API) and in used suspension concentrations in the study.

API	Concentration (mg/mL)	Pharmaceutical Class	Vehicle
Amoxicillin trihydrate	50	Antibiotic	SyrSpend^®^ SF PH4 liquid
Clozapine	25	Atypical antipsychotic	SyrSpend^®^ SF PH4 liquid
Indomethacin	5	Analgesic	SyrSpend^®^ SF PH4 NEO
Levodopa/ Carbidopa	10/ 2.5	Central nervous system agent/decarboxylase inhibitor	SyrSpend^®^ SF PH4 liquid
Levothyroxine Sodium (T4)	0.025	Hormone	SyrSpend^®^ SF PH4 liquid
Lomustine	4 and 10	Alkylating agents	SyrSpend^®^ SF PH4 liquid
Methyldopa	50	Antihypertensives	SyrSpend^®^ SF PH4 liquid
Procarbazine	10	Alkylating agents (malignancies)	SyrSpend^®^ SF PH4 liquid

**Table 2:** Chromatographic conditions used in the compatibility study.

API	Mobile Phase Composition (*v/v*)	Work Concentration (μg/mL) ^a^/Injection Volume (µL)	Column	Flux (mL/min)	Ultraviolet Detection Wavelength (nm)
Clozapine	Methanol:triethylamine:water (800:0.75:200)	100/10	C8, 4.6 mm × 250 mm at 25 °C	1.0	257
Indomethacin	Acetonitrile (550:450 *v*/*v*) with pH set to 8.0 with sodium hydroxide 1M	500/0.5	C8, 2.1 mm × 100 mm at 30 °C	0.3	290
Levodopa/Carbidopa	Alcohol:Buffer monobasic sodium phosphate pH 2.2 (5:95)	250/20	C18, 4.6 mm × 250 mm at 25 °C	1.0	280
Levothyroxine Sodium (T4)	750 mL of ultra-purified water + 2 mL of phosphoric acid:acetonitrile (70:30)	5/50	L10, 4.6 mm × 250 mm at 25 °C	1.0	225
Lomustine	Acetonitrile:water (1:1)	100/20	C18, 4.6 mm × 250 mm at 40 °C	1.5	230
Methyldopa	Phosphate buffer pH 3.0:methanol (85:15)	500/20	C18, 4.6 mm × 250 mm at 25 °C	1.0	280
Procarbazine	0.1M Dibasic phosphate buffer pH 7.0:methanol:acetonitrile (810:90:100).	100/20	C18, 4.6 mm × 250 mm at 25 °C	1.0	254

^a^ Diluted with mobile phase, unless specified otherwise.

**Table 3:** Summary of validation results of the HPLC/UHPLC methods.

APIs	Linearity						Specificity	Precision		Accuracy
	Range (µg/mL)	Analytical Curve	R^2^	F	LOD (µg/mL)	LOQ (µg/mL)	Discrepancy (%)	Repeatability (CV, %)	Intermediate Precision (CV, %)	Recovery (%)
Clozapine	70.07-130.13	y = 583841x − 1086406	0.9996	15719.29	0.005	0.014	1.98	0.12	2.05	100.19
Indomethacin	359.80-668.20	y = 0.039x + 0.0646	0.9978	3009.17	0.43	1.30	1.02	1.47	3.83	99.73
Levodopa/ Carbidopa	175.56-326.04	y = 15.92x − 271.22	0.9937	2041.11	0.01	0.03	0.56	0.17	3.22	100.54
Levothyroxine Sodium (T4)	3.52-6.54	y = 56.01x − 41.64	0.9910	712.06	0.02	0.06	1.68	0.81	0.77	101.62
Lomustine	70.56-131.04	y = 14.941x − 30.711	0.9968	2004.18	0.14	0.43	1.39	0.21	4.13	100.03
Methyldopa	350.07-650.13	y = 239370x − 4717139	0.9994	10018.78	0.0144	0.043	0.453	0.32	0.75	99.70
Procarbazine	72.52-134.68	y = 30.711 + 14.941	0.9991	6987.13	0.0003	0.001	1.94	0.71	0.61	100.05

CV, coefficient of variation; LOD, limit of detection; LOQ, limit of quantification. The acceptance criteria were: R^2^ > 0.99; F (significance of regression) >> 4.67; discrepancy < 2%; repeatability and intermediate precision < 5% and recovery = 100% ± 2% [18,19]. All analytical ranges were considered adequate to analyze the concentrations used.

**Table 4:** Summary of the stability- indicating study for the API.s.

API	HCl	NaOH	UV	Heat	H_2_O_2_
	%d *	%d	%d	%d	%d
Clozapine	−11.96	−85.17	−50.76	−5.11	−8.11
Indomethacin	−66.24	−100.00	−5.28	−12.35	−2.09
Levodopa/Carbidopa	1.38/4.21	0.43/−28.56	−0.23/2.74	1.69/3.31	−0.87/−1.59
Levothyroxine Sodium (T4)	−59.08	−58.34	−51.32	−80.02	−99.95
Lomustine	−99.66	−99.02	−29.57	−99.64	6.53
Methyldopa	−0.29	−97.84	−0.34	−3.35	−0.89
Procarbazine	−35.30	−72.24	−28.19	−70.79	−98.86

Results are presented as the average of three replicates, at three working concentrations. * %d = percentage of discrepancy between the active pharmaceutical ingredient peak without submission to stressing factors (negative control) and the peak of a sample subjected to one of the cited accelerated-degradation factors. Maximum acceptable = 2% (values higher than this are in bold). UV = ultraviolet.

**Table 5:** Stability of the active pharmaceutical ingredients in SyrSpend^®^ SF PH4.

Elapsed Time (Days)	%Recovery			
	Refrigerated Temperature (2–8 °C)		Controlled Room Temperature (20–25 °C)	
Amoxicillin 50 mg/mL				
T = 0	100.00 ± 0.07		100.00 ± 0.07	
T = 7	101.07 ± 0.23		99.37 ± 0.37	
T = 14	99.11 ± 0.05		97.41 ± 0.11	
T = 30	101.40 ± 0.02		94.38 ± 0.02	
Clozapine 25 mg/mL				
T = 0	100 ± 0.22		100 ± 0.22	
T = 7	99.98 ± 0.60		98.59 ± 0.45	
T = 14	98.54 ± 0.31		98.96 ± 0.42	
T = 30	99.03 ± 0.18		99.26 ± 0.45	
T = 60	99.21 ± 0.45		97.05 ± 0.34	
T = 90	99.39 ± 0.17		96.27 ± 0.42	
Indomethacin 5 mg/mL				
T = 0	100.00 ± 0.35		100.00 ± 1.66	
T = 7	99.93 ± 0.05		99.24 ± 1.01	
T = 14	98.49 ± 0.11		98.86 ± 0.03	
T = 30	95.56 ± 0.03		99.27 ± 0.07	
T = 60	95.48 ± 0.04		100.85 ± 0.03	
T = 90	95.81 ± 0.07		100.78 ± 0.08	
Levodopa 10 mg/mL + Carbidopa 2.5 mg/mL				
	Levodopa	Carbidopa	Levodopa	Carbidopa
T = 0	100.00 ± 2.38	100.00 ± 0.84	100.00 ± 2.38	100.00 ± 0.84
T = 7	101.69 ± 0.28	100.06 ± 0.90	98.36 ± 0.37	98.11 ± 1.78
T = 14	100.38 ± 1.77	99.46 ± 0.78	98.06 ± 0.14	97.80 ± 0.03
T = 30	100.54 ± 0.96	98.39± 0.69	98.88 ± 0.07	97.82 ± 0.02
T = 60	100.72 ± 0.55	98.91 ± 0.87	98.93± 0.09	97.88 ± 0.04
T = 90	100.63 ± 0.54	98.79 ± 1.03	98.44 ± 0.31	97.83 ± 0.26
Levothyroxine Sodium (T4) 0.025 mg/mL				
T = 0	100.0 ± 0.55		NP	
T = 7	96.52 ± 0.20		NP	
T = 14	96.26 ± 0.55		NP	
T = 30	96.22 ± 0.55		NP	
T = 60	96.00 ± 1.4		NP	
T = 90	96.06 ± 0.87		NP	
Lomustine 4 mg/mL				
T = 0	100 ± 0,29		NP	
T = 7	97.41 ± 0.31		NP	
T = 14	97.29 ± 0.17		NP	
T = 30	97.35 ± 0.19		NP	
T = 60	97.24 ± 0.88		NP	
T = 90	97.15 ± 0.34		NP	
Lomustine 10 mg/mL				
T = 0	100 ± 0.34		NP	
T = 7	99.33 ± 0.22		NP	
T = 14	98.55 ± 0.17		NP	
T = 30	99.13 ± 0.19		NP	
T = 60	98.63 ± 0.94		NP	
T = 90	98.57 ± 0.13		NP	
Methyldopa 50 mg/mL				
T = 0	100.00 ± 0.2		NP	
T = 7	99.17 ± 0.14		NP	
T = 14	99.81 ± 0.42		NP	
T = 30	99.35 ± 0.44		NP	
T = 60	99.44 ± 0.2		NP	
T = 90	99.52 ± 0.42		NP	
Procarbazine 10 mg/mL				
T = 0	100.00 ± 0.23		NP	
T = 7	99.75 ± 1.43		NP	
T = 14	100.10 ± 0.42		NP	
T = 30	100.67 ± 1.22		NP	
T = 60	80.44 ± 1.23		NP	

NP = not performed.

## Data Availability

There is no data availability to share.
